# Utilizing Artificial Intelligence to Predict Psychiatric Disorders in Patients with Inflammatory Bowel Disease (IBD): Insights Based on a Systematic Review

**DOI:** 10.1192/j.eurpsy.2025.1218

**Published:** 2025-08-26

**Authors:** A. A. Pillai, A. S. Nagendrapandian, J. Parmar, R. Walwaikar, A. A. Kumar, A. Agrawal, F. Sheikh

**Affiliations:** 1 SSPM Medical College and Lifetime Hospital, Sindhudurg, India; 2 West Windsor Plainsboro High School South, New Jersy, United States; 3 Government Medical College, Amritsar; 4 GMERS Medical College, Vadnagar, India; 5 Humanitas University, Milan, Italy; 6 Greater manchester mental health trust, Manchester, United Kingdom

## Abstract

**Introduction:**

The scientific literature recognizes the Gut-brain axis (GBA) as a crucial connection between gastrointestinal health and mental well-being. Patients with inflammatory bowel disease (IBD) are at a disproportionately higher risk of developing psychiatric disorders due to factors including gut dysbiosis and chronic inflammatory changes. Recent developments in artificial intelligence (AI) and machine learning, provide novel opportunities to predict the comorbid psychiatric outcomes in patients with IBD by analyzing complex datasets including but not limited to the gut microbiome and neuroimaging data.

**Objectives:**

This systematic review discusses the current evidence for AI-driven models to aid in the prediction of psychiatric disorders in IBD patients, with a focus on their performance and potential challenges around their clinical implementation.

**Methods:**

A systematic search on PubMed, EMBASE, Scopus, and Cochrane databases, identified 28 studies utilizing AI-based models to examine gut microbiota and neuroimaging data in patients with IBD. Data extraction illuminated the following artifacts: classification thresholds (i.e. predictive), relevant supervised learning or deep learning modeling (e.g. random forest classifiers, convolutional neural networks, and unsupervised models like attention-based learning), sensitivity, specificity, accuracy, and both accuracy measures and AUC-ROC curve values.

**Results:**

A pooled analysis of the included studies demonstrated an estimated sensitivity of 81% (95% CI: 77-85%) and specificity of 78% (95% CI: 73-82%) to predict psychiatric disorders in patients with IBD with the highest predictive accuracy elicited by studies based on microbiome and neuroimaging data. Yun et al. (2024), for instance, demonstrated a predictive accuracy of 86% using microbiome profiles and structural brain imaging data while Fil et al. (2024) elucidated the positive correlation between gut dysbiosis and psychiatric symptoms based on microbial signature models. Additionally, the variability noted in the predictive performance of the models was found to be based on the patient population, quality of data, and machine learning strategy.

**Conclusions:**

AI models present promising evidence in predicting psychiatric disorders in IBD patients by leveraging microbiome and neuroimaging datasets. Overall, the meta-analysis reports strong predictive strength with high sensitivity and specificity. Future work in this field should focus on the validation of these prediction models in various clinical populations, improving their generalizability and standardization to enable widespread use and integration in the field of personalized psychiatry, especially in patients with IBD.

**Disclosure of Interest:**

None Declared

